# Sequential Dose-Dense Doxorubicin and Ifosfamide in Advanced Soft-Tissue Sarcoma Patients in an Out-Patient-Basis Schedule

**DOI:** 10.1155/2011/984340

**Published:** 2011-06-30

**Authors:** G. F. G. Almeida, G. Castro, I. M. L. Snitcovsky, S. A. Siqueira, E. H. Akaishi, O. P. Camargo, C. R. G. C. M. Oliveira, M. H. H. Federico

**Affiliations:** ^1^Servico de Oncologia Clinica, Instituto de Radiologia, Hospital das Clinicas, Faculdade de Medicina, Universidade de Sao Paulo, 05403-900 Sao Paulo, SP, Brazil; ^2^Centro de Hematologia e Oncologia—CEHON, Avenida Araujo Pinho, 439 Canela, 40110-150 Salvador, BA, Brazil; ^3^Servico de Anatomia Patologica, Hospital das Clinicas, Faculdade de Medicina, Universidade de Sao Paulo, 05403-010 Sao Paulo, SP, Brazil; ^4^Terceira Clinica Cirurgica, Hospital das Clinicas, Faculdade de Medicina, Universidade de Sao Paulo, 05403-010 Sao Paulo, SP, Brazil; ^5^Instituto de Ortopedia e Traumatologia, Hospital das Clinicas, Faculdade de Medicina, Universidade de Sao Paulo, 05403-010 Sao Paulo, SP, Brazil; ^6^Laboratorio de Anatomia Patologica, Instituto de Ortopedia e Traumatologia, Hospital das Clinicas, Faculdade de Medicina, Universidade de Sao Paulo, 05403-010 Sao Paulo, SP, Brazil

## Abstract

*Aims*. This phase II study explored activity/safety of front-line dose-dense chemotherapy in high-grade STS (soft tissue sarcoma) patients and tested ezrin as prognostic factor. *Patients and Methods*. The protocol consisted of three cycles of doxorubicin (DOXO) 30 mg/m^2^ on days 1–3 every 2 weeks, followed by three cycles of ifosfamide (IFO) 2.5 g/m^2^ two hours a day on days 1–5 every 3 weeks, with GCSF support. Ezrin was assessed immunohistochemically. *Results*. Twenty patients, 13 metastatic and 7 locally advanced, were enrolled. Median age was 39 years (25–60). Median dose intensities were 42 mg/m^2^/week and 3.6 g/m^2^/week for DOXO and IFO, respectively. Grade 3/4 toxicities occurred in 18 patients. Response rate was 15% (3 of 20) by RECIST. Patients younger than 45 years with locally advanced disease and synovial histology presented longer survival. A trend towards longer survival was observed among ezrin-positive patients. *Conclusions*. This dose-dense schedule should not be routinely used due to its high frequency of toxic events; however, a sequential strategy with DOXO and IFO may benefit selected patients and should be further explored with lower doses. The role of ezrin as a prognostic marker should be confirmed in a larger group of patients.

## 1. Introduction

High-grade, advanced, soft-tissue sarcoma (STS) is an aggressive disease with poor prognosis. In this population, single-agent doxorubicin (DOXO) has been considered the standard in detriment of combination regimens [1]. On the other hand, in view of the fact that STS patients maintain response to ifosfamide (IFO) after DOXO failure [2], the sequential regimen emerges as a useful tool in an attempt to enhance treatment efficacy through better compliance.

Testing this strategy, the first sequential (dose dense) STS study was shown to be feasible with manageable toxicity [3]. The second one, nonetheless, observed no survival advantage over single-agent DOXO [4]. However, unselected patient population might have obscured a potential benefit from the use of DOXO and IFO. Supporting this idea, a meta-analysis of adjuvant chemotherapy in STS has shown that survival benefit was restricted to patients exposed to both agents [5]. Therefore, it is essential to identify those patients in whom this strategy is worthwhile, particularly the ones with higher risk of death. 

In this scenario, one STS trial has demonstrated that patients with positive ezrin expression presented poor survival [6]. Ezrin is a member of the ERM (ezrin/radixin/moesin) family of cytoskeleton-associated proteins [7] involved in cell to the extracellular matrix, as well as in cell-cell interactions, receptor tyrosine-kinase signaling, transduction, and interactions with apoptotic machinery [8, 9]. Enhanced ezrin expression had been correlated with clinical stage and metastatic behavior of rhabdomyosarcoma cells [10]. In another study, ezrin was found to be necessary for metastasis and survival in a mouse model of osteosarcoma [11]. 

The present study explored the activity and safety of front-line sequential dose-dense DOXO- and IFO-based chemotherapy in advanced, high-grade STS adult patients in an outpatient basis, and we tried to confirm if ezrin could identify those patients with worse prognosis. The primary endpoint of the trial, response rate, was evaluated by RECIST version 1.0 [12]. Secondary endpoints were overall survival, progression-free survival, safety, and treatment compliance. Toxicity profile was categorized according to the National Cancer Institute Common Toxicity Criteria version 2.0.

## 2. Patients and Methods

### 2.1. Patient Selection

This study included metastatic (META) or locally advanced (LOCA), chemotherapy-naïve, histologically confirmed, high-grade STS patients according to the WHO classification. Patients were deemed eligible who were 18–60 years of age, had at least one unidimensionally measurable lesion, an ECOG performance status of 0–2, adequate bone marrow, and renal, liver, and cardiac functions (neutrophils count >1.0 × 10^9^ per liter and left ventricle ejection fraction >55%). Patients with malignant mesothelioma, juvenile types of rhabdomyosarcoma, neuroblastoma, PNET/Ewing, carcinosarcoma, and gastrointestinal stromal tumor were excluded.

The protocol was reviewed and approved by the Ethics Committee for Research Project Analysis from University of Sao Paulo, and conducted according to the International Conference on Harmonization Good Clinical Practice guideline. All participants were advised about the procedures and gave informed consent.

### 2.2. Trial Design

As cited previously, all chemotherapy was administered on an outpatient basis. Three cycles of 30 mg/m^2^/day* in bolus* DOXO on days 1–3, q2w, were followed by three cycles of two-hour infusions of 2.5 g/m^2^/day IFO on days 1–5, q3w. Mesna equimolar to IFO was administered in two half doses given immediately before and 4 hours after IFO. Each cycle was followed by G-CSF 300 *μ*g/day for 7 days. Echocardiograms and scanned images of the primary tumor and metastatic lesions (CT and/or MRI) were taken before treatment, between DOXO and IFO cycles, and after the final IFO cycle. Dosage was reduced 20% whenever nonhematological grade 3/4 or hematological grade 4 toxicity occurred.

### 2.3. Statistical Design

This trial required 39 patients to achieve 80% statistical power at a significance level of   *P* < .05 in detecting a 20% increase from the expected response rate of 30%. With the minimax two-step design [13], six responses among the first 19 patients were required in order to accrual the entire cohort. All subgroup analyses were univariate due to the limited number of patients included in this trial.

### 2.4. Ezrin Expression

Ezrin was analyzed immunohistochemically on formalin-fixed, paraffin-embedded biopsy slides as described elsewhere [6]. Briefly, sections were incubated overnight with antiezrin antibody (clone 3C12, NeoMarkers), and they were then exposed to biotinylated secondary antibody and avidin-biotin-peroxidase (LSAB, K690, Dako). Slides from placenta served as controls using the same procedures with and without the primary antibody. Cytoplasmic immunostaining in least 1% of tumor cells was scored as positive.

## 3. Results

### 3.1. Patient Characteristics

**Figure 1:**
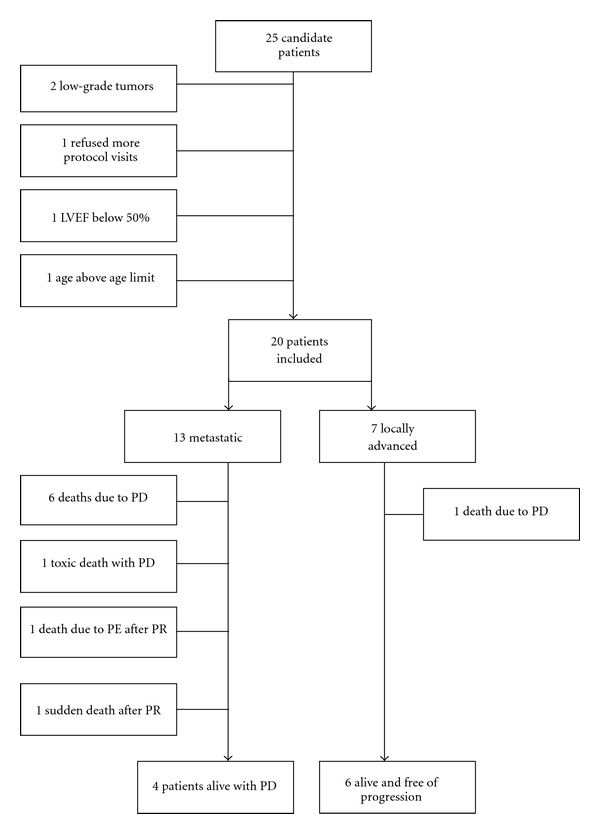
Patient inclusion. LVEF: left ventricular ejection fraction. PE: pulmonary embolism. PR: partial response. PD: progressive disease.

In this uni-institutional, single-arm phase II study, 25 patients with high-grade STS were screened between August 2005 and January 2007, and 20 entered (Figure 1). Thirteen patients had META disease and seven had LOCA disease (five of these LOCA patients presented with neurovascular involvement; Table 1). The median length of time from initial diagnosis until inclusion in the trial was 2.4 months (0.6–30 months).

### 3.2. Treatment Compliance

In total, 105 cycles were delivered, 54 during the first phase and 51 during the second. Fourteen patients (70%) received all planned chemotherapy. Six META patients did not complete the protocol; this was due to disease progression in three patients, two early deaths (see discussion below), and in one patient DOXO was halted after a single cycle due to unexplained cough. This patient presented a pretreatment left ventricle ejection fraction (LVEF) of 58% and diffuse myocardial hypocontractility. He underwent a new LVEF evaluation, which remained unchanged; he became asymptomatic and received six IFO cycles at a dose of 10 g/m^2^ at the investigators' discretion. He presented stable disease and underwent resection of lung metastases. 

A median of six cycles (1–7) were administered per patient. Median dose intensities for DOXO and IFO for the entire cohort were 42 mg/m^2^/week (27–45) and 3.6 g/m^2^/week (1.3–4.1), respectively, which corresponded to 93% (61–101%) and 87% (32–100%) of the planned dose intensities.

### 3.3. Objective Response

In an intention-to-treat analysis, we observed three partial responses, 10 stable diseases, and seven disease progressions, translating to a response rate of 15% (3 out of 20). As mentioned previously, because this fell short of the six responses required to complete accrual, the study closed accrual on January 31st 2007. The histologic subtypes of the patients who presented partial responses were synovial sarcoma, unclassified sarcoma, and malignant fibrous histiocytoma (MFH). Median duration of response was 2.1 months (1.3–4.9).

### 3.4. Progression-Free and Overall Survival

Survival information was locked on August 31st 2007. After a median follow-up of 11 months with 10 patients remaining alive, progression-free survival (PFS) and overall survival (OS) were 8.1 months (1.5–22.6) and 20.1 months (2.3–22.6), respectively. Subgroup analyses showed longer PFS for those patients in the LOCA group as compared to META patients, not reached versus 6 months, HR 0.07 (95% CI 0.04–0.43) *P* = .0005. OS was also longer among LOCA versus META patients, not reached versus 8.6 months, HR 0.12 (95% CI 0.06–0.78) *P* = .01. No synovial sarcoma patients had died at the time of survival analysis which translated to longer survival in comparison to other histologies, OS not reached versus 14.2 months, HR 0.00 (95% CI 0.05–0.78) *P* = .02. The same was true for those patients younger versus older than 45 years old, OS 20.1 months versus 4.2 months, HR 0.32 (95% CI 0.03–0.99) *P* = .04.

### 3.5. Adverse Events, Serious Adverse Events and Deaths

The toxicities that occurred in more than 10 cycles are detailed in Table 2. Grade 3–4 toxicities, predominantly hematological, occurred in 76 of 105 cycles. Only one nonhaematologic grade 4 toxicity (colitis) was observed. One grade 1 and one grade 2 spontaneously reversible encephalopathies were observed. No creatinine elevation was observed; however, one IFO-inducible grade 3 haematuria occurred, requiring dose reduction as previously described. A single toxic death occurred in a 60-year-old patient with an MFH primary of the shoulder, who died due to grade 4 colitis 1.9 month after the third DOXO cycle. Three early deaths occurred without signs of toxicity: the first was a 59-year-old patient with an unclassified sarcoma primary of the pelvis who died as a consequence of lung metastases after receiving one DOXO cycle; the second was a 58-year-old patient with MFH primary of the thigh who died suddenly within three weeks after completing the protocol; and the third was a 47-year-old patient with an unclassified sarcoma primary of the lung who died of pulmonary embolism after one IFO cycle. Five other patients presented nonfatal thromboembolic complications: two grade 3 deep vein thromboses (DVT), one grade 4 pulmonary embolism in the META group, and two grade 3 DVT in the LOCA group. All DVT cases were documented in the primary lesion limb. Among 16 evaluable patients, three presented cardiotoxicity, two grade 1, and one grade 3.

### 3.6. Ezrin Expression

**Figure 2:**
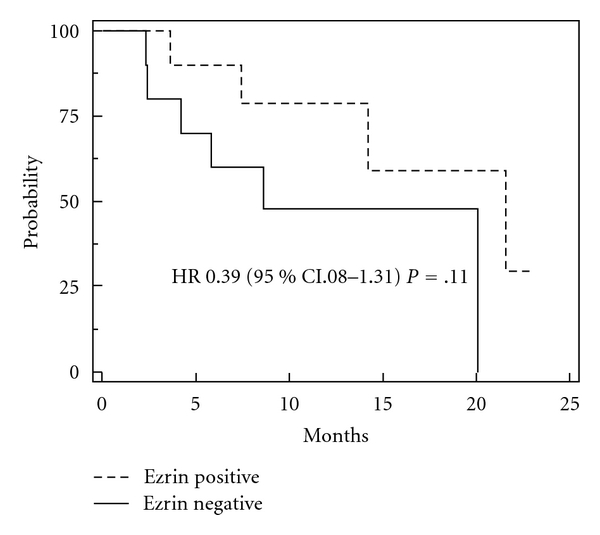
Overall survival according to immunohistochemical ezrin expression. Median overall survival for soft-tissue sarcomas patients whose tumors were classified as positive for ezrin was 21.1 months, compared to 8.6 months for ezrin-negative patients; HR 0.39, 95% CI 0.08–1.31; *P* = .11.

Ezrin expression was positive in 10 patients (Table 3). There was no correlation between ezrin positivity and either response or presence of necrosis. Unexpectedly, all five synovial sarcoma patients were positive for ezrin, correlation that has demonstrated to be statistically significant (Fisher's exact test, *P* = .03). As illustrated in Figure 2, a trend toward longer survival was observed among ezrin positive versus negative patients, 21.1 months versus 8.6 months, HR 0.39 (95% CI 0.08–1.31) *P* = .11.

## 4. Discussions

In this phase II study investigating a sequential dose-dense DOXO/IFO schedule in high-grade STS patients, a low response rate has been observed, determining patients' accrual discontinuation. Unexpectedly, none of the LOCA patients responded; however, all but one was alive at the time of study closure. On the other hand, two of the three patients presenting partial response had died, raising the question whether response rate will represent an appropriate endpoint in future STS trials.

Despite a high degree of grade 3/4 toxicities observed, febrile neutropenia rate was similar to that observed in the literature [14], and this occurred irrespective of the lower neutrophil threshold and less GCSF support [3, 14]. GCSF use has been implicated in poor outcome [15]. The incidence of cardiotoxicity was not higher than that observed in the literature [3, 14]; however, a great number of thromboembolic complications may suggest that mechanical factors and changes in procoagulant systems after chemotherapy [16] might have been boosted by dose density and should be used with caution. IFO administration over 2 hours in five days may result in less renal and neurologic complications. All these nuances have practical implications.

The median OS was longer than those described in two previous studies with similar dose-dense strategies [3, 4]. We have included more resectable patients, more synovial histology, and we have excluded subtypes such as carcinosarcoma and clear cell sarcoma which are known to be, respectively, sensitive to cisplatin [17] (absent in this protocol) or irresponsive to systemic treatment [18]. Additionally, in one of the earlier trials [4], prophylactic use of erythropoietin may have detrimentally influenced OS [19]. Statistical limitations, along with heterogeneity of patient populations, preclude drawing firm conclusions through direct comparison to these dose-dense studies. Nevertheless, we have found selected patients who presented longer survival in our population, nonetheless we know that this would unlikely hold up on a multivariate analysis, underpowered because of small number of patients. If this was due to this sequential approach using both agents DOXO and IFO may be considered an issue of debate. 

In a scenario of doubts in which STS trials are inserted, some questions arise. (1) Are there patients who might benefit from DOXO and IFO in first-line chemotherapy? (2) Are these patients among the ones with poor prognosis? (3) How could we find them? The answer for the first two questions is “perhaps” and for the third one, we will use selected histologic subtypes as examples. Leiomyosarcoma patients may show response rate (RR) as low as zero [20], in contrast to synovial sarcoma ones that may be especially sensitive to ifosfamide with RR as high as 100% [21]. In this setting, tumor size and grade have been considered the most important prognostic factors; however, even those patients with the same histology-size-grade-tumors experience discrepant survivals. In an effort to reduce this limitation, the incorporation of gene expression analyses has permitted the improvement in diagnosis [22], prognosis prediction [23–25], and the identification of new molecular targets that may be used for the development of new drugs [26]. The new era of molecular-target-driven treatment has shown that survival benefit is missed when unselected patient population is treated. This is undoubtedly applicable for soft-tissue sarcoma trials.

We have observed that, besides being 100% ezrin positive, those patients with synovial histology had most often locally advanced disease (with a better prognosis than metastatic disease), and we believe that this clearly confounds this data. However, we also believe that ezrin may have positively influenced survival among those patients. Corroborating these results, a better outcome was also observed for patients with ovarian cancer and positive for ezrin [27]. This is in contrast to the earlier STS report [6]; however, a possible explanation for this latter discrepancy may be the approximately 70% malignant fibrohistiocytoma histology comprising that study population [6]. In agreement with our results, the only patient with synovial histology in the earlier trial who was ezrin-positive presented 16 months OS, an outcome that may be considered favorable. Moreover, one ezrin-positive leiomyosarcoma patient included in our study had 14.2 month OS, compared to the median 8.2 months of four ezrin-negative ones. This raises the hypothesis of a differential role played by ezrin in each STS histology. The role of ezrin as a prognostic indicator should be confirmed in future prospective STS trials in larger populations. 

In accordance to the literature [4], we conclude that sequential dose-dense DOXO- and IFO-based chemotherapy does not appear suitable for routine treatment of high-grade advanced STS patients. However, this sequential strategy with DOXO followed by a two-hour ifosfamide regimen does warrant further investigation in lower doses, trying to find the balance between efficacy and safety.

The fact that STS are heterogeneous and cannot be grouped and treated as the same way is the new paradigm. In the near future, the identification of one (or more) biologic (molecular) marker(s) will tailor treatment in an attempt to predict compliance, response, and survival.

## Figures and Tables

**Table 1:** Patient characteristics.

	META^a^	LOCA^b^	Total
	*N *(%)	*N* (%)	*N* (%)
Total	13 (65)	7 (35)	20 (100)

Sex			
Male	7 (35)	5 (25)	12 (60)
Female	6 (30)	2 (10)	8 (40)

Age (years)			
Median	47	28	39
Range	26–60	25–45	25–60

Performance status			
0	4	4	8
1	6	3	9
2	3	0	3

Histological subtype			
Synovial	1	4	5
Leiomyo	5	0	5
Unclassified	2	2	4
Malignant fibrohistiocytoma	2	0	2
Myxoid	1	1	2
Other^c^	1	1	2

Primary site			
Lower limbs	3	5	8
Retroperitoneal	5	0	5
Upper limbs	2	2	4
Other^d^	3	0	3

Size of primary tumor (cm)			
Median	12.4	11.7	12
Range	2–18	6.1–15	2–18

Metastatic site			
Lung	11	NA	11
Lymph node	5	NA	5
Liver	4	NA	4
Bone	4	NA	4
Other^e^	4	NA	4

Number of metastatic sites			
1	5	NA	5
2	3	NA	3
≥3	5	NA	5

^a^META: metastatic disease.

^b^LOCA: locally advanced disease.

^c^Epithelioid, malignant peripheral nerve sheath tumor.

^d^Lung, uterus, scalp.

^e^Spleen, adrenal gland, pleura.

NA: not applicable.

**Table 2:** Adverse events occurring in more than 10 cycles.

Event	Grade 1–4-related events (*N*)			
	1	2	3	4
*Nonhaematological*				
Alopecia	7	12	NA	NA
Anorexia	6	6	0	0
Asthenia	1	16	4	0
Canalicular enzymes	34	5	2	0
Constipation	5	16	1	0
Dermatitis	9	5	0	0
Diarrhea	7	1	3	0
Dysgeusia	8	5	0	0
Haematuria	12	2	2	0
Hypobicarbonatemia	13	0	0	0
Mucositis	17	12	3	0
Nausea	23	15	3	0
Transaminitis	18	3	1	0
Vomiting	20	8	6	0

*Hematological*				
Anemia	41	20	5	0
Thrombocytopenia	7	3	2	0
Febrile Neutropenia	NA	NA	5	1

NA: not applicable.

**Table 3:** Ezrin expression according to histology.

Histology	Ezrin expression
Leiomyosarcoma	+++
Leiomyosarcoma	0
Leiomyosarcoma	0
Leiomyosarcoma	0
Leiomyosarcoma	0
Synovial	++++
Synovial	+++
Synovial	++
Synovial	+
Synovial	+
Unclassified	++
Unclassified	0
Unclassified	0
Unclassified	0
MFH^a^	+
MFH^a^	0
Myxoid	+++
Myxoid	0
Epithelioid	+
MPNST^b^	0

^a^Malignant fibrohistiocytoma.

^b^Malignant peripheral nerve sheath tumor.
